# The Dean of St. Paul's on Eugenics

**Published:** 1920-11-27

**Authors:** 


### The Dean of St. Paul's on Eugenics.

It is impossible to report adequately the lecture on Eugenics and Religion recently given by the Dean of St Paul's at the Wigmore Hall. He maintained that the partial failure of the Eugenist Society was not due chiefly to the unbending conservatism of religion, but to the sentimentality of the public. Secular people, as much as religious people, were found taking no interest in eugenics; politicians were notably absolutely uninterested in racial development, perhaps because the unborn had no vote. At present the whole nation, especially the Government, is behaving as if we have made a fortune in the war.

Eugenists do not believe that there is any natural or supernatural power which will intervene to save us from the consequences of transgressing the laws of Nature. There is nothing in the Christian religion to foster irrational emotionalism, which is opposed to science. Eugenists have no fixed dogmas, and would even dissolve the Society if science proved that they were on the wrong lines. But the man who says that birth control should be forbidden, and does not admit that the consequences of this prohibition will be poverty and degeneration, is the man who angers the eugenist.

If rational selection does not take the place of natural selection, the country will deteriorate. It is an age producing no great men—no great poets, artists, statesmen, or scientists. The much-abused Victorian age brought us great men, men who will be great to posterity in spite of the opinions of Mr. Lytton Strachey and his like.

A bishop had been known to declare that the eugenist encouraged brawn before brain. That was not so; but, all the same, Dr. Inge hoped that the brawn would thrive, for the athlete was a beautiful creature.

"We have encouraged the misfits to multiply," he said; "we breed from our worst stock, and our best is being squeezed out of existence." The degenerates breed most among the lower quarters; they tend to die out in higher circles unless there is great beauty, great wealth, or a title as a makeweight.

Speaking of the present day, Dr. Inge said when the power lay with the untaxed class the end was the bankruptcy of the nation. There was the extravagance of the untaxed Kings of France which had led to the French Revolution, while on the other hand, when the middle class in the last era had paid the taxes and retained the power, the country was prosperous and economical. Eventually the present wastefulness would come to an end, because there would be nothing left to waste.

In conclusion, Dr. Inge said he was afraid that the future would see many childless and servantless homes among the professional classes. He hoped that religion might help eugenics by teaching that the poverty which children might entail was bearable if the mind was set on higher things, on all that was pure and good and true. He knew that families would have to be small, but, at the same time, he hoped the childless home would be an exception.

